# Reproducibility of microvessel counts in breast cancer specimens

**DOI:** 10.1038/sj.bjc.6690811

**Published:** 1999-11

**Authors:** L P Marson, K M Kurian, W R Miller, J M Dixon

**Affiliations:** Edinburgh Breast Unit, Breast Unit Research Group andDepartment of Pathology, Paderewski Building, Western General Hospital, Crewe Road, Edinburgh EH4 2XU, UK

**Keywords:** core biopsy, microvessel count, angiogenesis

## Abstract

Assessment of tumour vascularity in core biopsy specimens may be a useful predictor of response to primary therapy. This study addresses practical methodological issues regarding accuracy of tumour vascularity assessments in different breast cancer specimens. Issues addressed in the study are variation caused by (i) inherent observer variation in the method, (ii) tumour heterogeneity and (iii) previous surgical manipulation of tumours. Microvessel counts were performed by two observers on separate occasions and by two different observers. Counts were performed on core biopsies and tumour sections taken simultaneously (*n* = 16) and with an intervening time interval (*n* = 21). In addition core biopsies were obtained from the same tumour on two separate occasions (*n* = 10). A highly significant correlation was found in counts performed by the same observers at different times and between two different observers. No significant correlation was found in counts of core biopsies and tumour sections taken either simultaneously or subsequently. No correlation was found between counts of sequential core biopsies. Study findings suggest that, although microvessel counts may be assessed reproducibly by the same and different observers, counts performed in core biopsies do not accurately reflect those of overall tumour, limiting their potential as predictive or prognostic markers. © 1999 Cancer Research Campaign


*British Journal of Cancer* (1999) **81**(6), 1088–1093
© 1999 Cancer Research Campaign
Article no. bjoc.1999.0811

### Reproducibility of microvessel counts in breast cancer specimens

**LP Marson[1], KM Kurian[3], WR Miller[2] and JM Dixon[1]**

[1]Edinburgh Breast Unit, [2]Breast Unit Research Group and [3]Department of Pathology, Paderewski Building, Western General Hospital, Crewe Road, Edinburgh EH4 2XU, UK

**Summary** Assessment of tumour vascularity in core biopsy specimens may be a useful predictor of response to primary therapy. This study addresses practical methodological issues regarding accuracy of tumour vascularity assessments in different breast cancer specimens. Issues addressed in the study are variation caused by (i) inherent observer variation in the method, (ii) tumour heterogeneity and (iii) previous surgical manipulation of tumours. Microvessel counts were performed by two observers on separate occasions and by two different observers. Counts were performed on core biopsies and tumour sections taken simultaneously ($n$ = 16) and with an intervening time interval ($n$ = 21). In addition core biopsies were obtained from the same tumour on two separate occasions ($n$ = 10). A highly significant correlation was found in counts performed by the same observers at different times and between two different observers. No significant correlation was found in counts of core biopsies and tumour sections taken either simultaneously or subsequently. No correlation was found between counts of sequential core biopsies. Study findings suggest that, although microvessel counts may be assessed reproducibly by the same and different observers, counts performed in core biopsies do not accurately reflect those of overall tumour, limiting their potential as predictive or prognostic markers. © 1999 Cancer Research Campaign

**Keywords**: core biopsy; microvessel count; angiogenesis

New vessel formation, or angiogenesis, is a pre-requisite for tumour growth beyond 1–2 mm$^3$ and for metastasis (Folkman, 1990, 1994). Assessment of angiogenesis in histological sections by counting tumour microvessels has been shown to provide independent prognostic information in the majority of studies in breast cancer (Weidner et al, 1991; Horak et al, 1992; Gasparini et al, 1994).

This study addresses practical issues relating to methodology. Not all studies have found microvessel counts to be of prognostic value (Van Hoef et al, 1993; Axelsson et al, 1995; Costello et al, 1995), which may be due in part to problems with reproducibility. Individual investigator experience affects results (Vermeulen et al, 1997) and other studies have found high rates of inter-observer variation (Van Hoef et al, 1993; Axelsson et al, 1995).

Heterogeneity of vasculature in breast tumours is well recognized (Weidner et al, 1991; Van Hoef et al, 1993) and has led to the recommendation of selection of areas of highest vascularity or 'hot spots' in which to perform microvessel counts. This is based on the rationale that it is the highly angiogenic tumour cell clones that are most likely to behave aggressively and to metastasize (Weidner et al, 1992; Vermeulen et al, 1996). A transverse tumour section has been found to be representative of whole tumour when microvessel counts in vascular 'hot spots' were compared with radiological grades from micro-angiographic assessments of the same tumours (Martin et al, 1997*b*). However, another study addressing similar issues found noteworthy heterogeneity of microvessel counts between different areas of the same tumour and emphasized the need for careful 'hot spot' selection (de Jong et al, 1995). Attempts have been made to improve objectivity of the counting method with use of a Chalkley eyepiece graticule or computerized image analysis (Fox et al, 1995).

As an increasing number of patients undergo primary systemic treatment prior to tumour excision, the only specimen available for studies of potentially useful prognostic and predictive markers are core needle biopsies. Such core biopsies provide useful histological information (Minkowitz et al, 1986), but such material may not be representative of overall tumour vascularity (Jacobs et al, 1998). In addition, effects of previous surgical manipulations on tumour vascularity assessment have not been addressed.

The aims of the present study were: (i) to assess intra-observer and inter-observer variation in microvessel counts; (ii) to determine whether core biopsies are representative of whole tumour; (iii) to investigate effects of previous surgical manipulation on subsequent biopsies.

### MATERIALS AND METHODS

### Patient material

#### *Core biopsies*

Biopsies were performed following infiltration of the area with 20 ml 1% lignocaine with 1:200 000 adrenaline via a single stab incision at a point lateral to the lesion. Between one and seven biopsies were performed using a Pro-Mag gun and 14-gauge needle.

#### *Tumour sections*

Immediately following surgical excision, complete transverse sections of tumour were collected, including central and peripheral tumour areas.

*Received 1 December 1998*
*Revised 18 March 1999*
*Accepted 20 April 1999*

*Correspondence to:* LP Marson

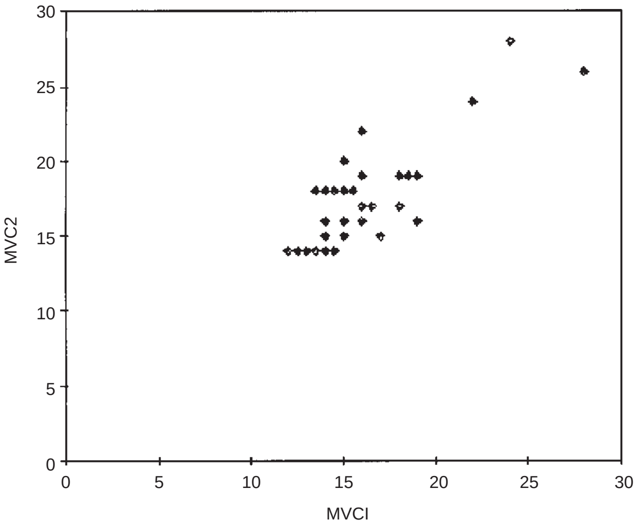

**Figure 1** Correlation of counts at different time points, where mvcl is the first count and mvc2 is the second (Spearman rank correlation: $r = 0.84$, $P < 0.001$)

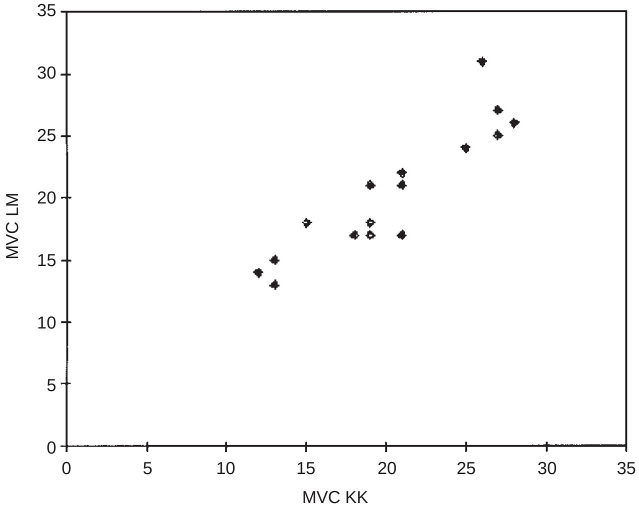

**Figure 2** Correlation of counts performed by different observers, where mvcKK is the count performed by Dr Kurian and mvcLM is the count performed by Miss Marson (Spearman rank correlation: $r = 0.83$, $P < 0.001$)

### Study design

Three comparative studies were performed:

1. In 16 excised tumours, core biopsies were taken and tumour sections obtained at the same time. This allowed direct comparison of counts performed on core biopsies and sections taken simultaneously.
2. Twenty-one patients with newly diagnosed breast cancer underwent core biopsies for confirmation of diagnosis and determination of oestrogen receptor status. Following surgical excision of tumour between 5 and 35 days later, transverse tumour sections were collected. This allowed comparison of counts performed on core biopsies and tumour sections taken at a later time with no intervening treatment.
3. In ten cases, core biopsies were performed at diagnosis and repeated immediately following surgical excision, between 5 and 25 days later, thus allowing comparison of counts on core biopsies from the same tumour taken at different times.

### Tumour preparation

All specimens were fixed in formalin for a minimum period of 24 h and paraffin-embedded. Four-micrometre sections were cut and a section of each sample stained with haematoxylin and eosin for histological assessment.

### Immunohistochemistry

Immunohistochemistry was performed following antigen retrieval with protease using anti-CD31 antibody (Dako) (Parums et al, 1990). The alkaline phosphatase anti-alkaline phosphatase (APAAP) technique was used to visualize antibody binding (Cordell et al, 1984).

### Microvessel counts

Following staining with CD31 the three areas of highest vascularity were identified by scanning sections at low power (×40 and ×100). Counts were performed at high power (×250) using a Chalkley 25-point eyepiece graticule. In these areas, the graticule was oriented so that the maximum number of points lay on or within areas of highlighted vessels. Microvessel count (mvc) was taken as the total of three counts. All counts were performed using a conference microscope with two observers, a pathologist (KK) and a surgeon (LM) who had undergone a period of training in the technique. In addition, the individual investigators performed separate counts on 16 tumours to assess interobserver variability and together repeated counts on 31 tumours at different times to assess intra-observer variation.

### Statistical analysis

Comparisons of pairs were made using Wilcoxon rank test. An expression of variation was calculated for intra- and inter-observer variation, as the standard deviation of the difference between two groups/mean of total. Correlations were determined using the Spearman rank correlation test.

### RESULTS

### Intra- and inter-observer variation

Microvessel counts performed by the same observers on two occasions (mvc1 and mvc2) at least a fortnight apart were compared in 31 tumours, of which 16 were core biopsies. Correlation between counts is illustrated in Figure 1. Mvc1 ranged from 12 to 28 (median: 16, mean: 16.4) and mvc2 from 14 to 28 (median: 17, mean: 17.7). There was significant correlation between the two counts ($r = 0.84$, $P < 0.001$). The standard deviation of difference between the two groups was 1.95, giving an expression of variation of 11%.

Microvessel counts were performed by two different observers (mvcKK and mvcLM) in 16 tumours, all of which were tumour sections. MvcKK ranged from 12 to 28 (median: 20, mean: 20.3), mvcLM ranged from 13 to 31 (median: 19.5, mean: 20.5) as shown in Figure 2. A significant correlation was found between

© 1999 Cancer Research Campaign

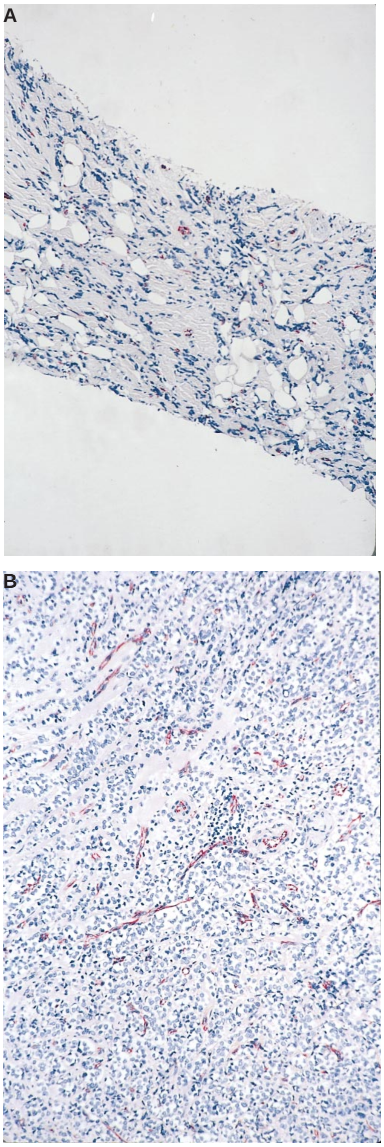

**Figure 3** (**A**) Illustration of immunohistochemical staining with antibody to CD31 performed on a core biopsy section. (**B**) Illustration of immunohistochemical staining with antibody to CD31 performed on a cross-section of the same tumour, with vessels highlighted in red against a background of haematoxylin counterstaining

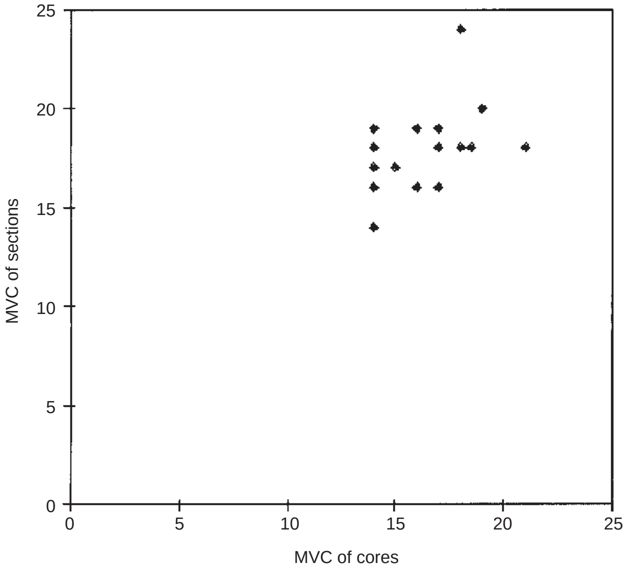

**Figure 4** Relationship between microvessel count of cores and tumour sections taken simultaneously (Spearman rank correlation: $r = 0.44$, $P = 0.09$)

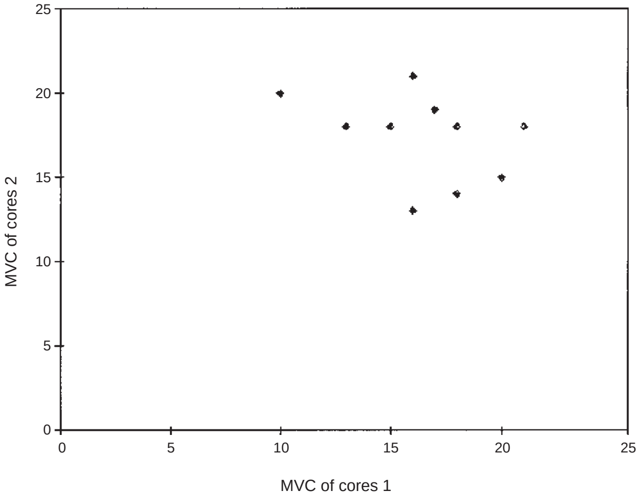

**Figure 5** Relationship between mvc of cores and subsequent tumour sections (Spearman rank correlation: $r = 0.1$, $P > 0.05$, paired Wilcoxon: $P = 0.0065$)

counts of the two observers ($r = 0.83$, $P < 0.001$). The standard deviation of differences was 3.03, giving an expression of variation of 15%.

### Comparison between mvc of core biopsies and tumour cross-section from excised tumours

Core biopsies were taken of 16 tumours immediately following surgical excision. The number of core biopsies taken from each tumour ranged from 1 to 7 (median: 3, mean: 3.3). Figure 3 illustrates immunohistochemical staining of vessels with antibody to CD31 in a core biopsy section (Figure 3A) and tumour section (Figure 3B).

Figure 4 illustrates the correlation between counts in cores and tumour sections. Mvc in core biopsies ranged from 13 to 22 (median: 17, mean: 16.5) and in tumour sections from 14 to 24 (median; 18, mean: 18). There was no significant difference in mvc between the two groups ($P = 0.1$, paired Wilcoxon). Conversely whilst there was a positive correlation in counts, this just failed to reach statistical significance ($r = 0.44$, $P = 0.09$). Mvc was higher in tumour sections than core biopsies in nine of 16 tumours (56%), was higher in core biopsies in three of 16 tumours (18.8%) and the same in four (25%).

### Comparison between mvc of core biopsy and subsequent tumour cross-section

Core biopsies were taken for diagnosis in 21 patients who underwent surgery between 5 and 34 days later (median time to surgery: 23 days), with no intervening treatment. One patient was found to have carcinoma in situ with no evidence of invasion and was excluded from the study. The number of core biopsies taken

 *© 1999 Cancer Research Campaign*

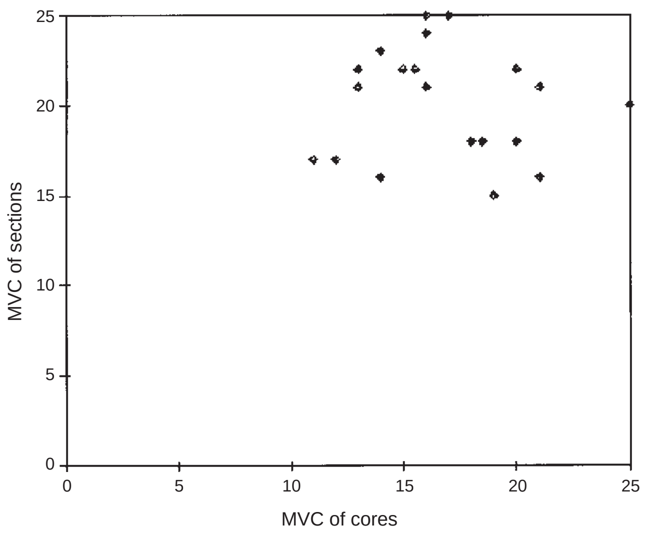

**Figure 6** Relationship between mvc performed on core biopsies taken at separate time points, where mvc of cores 1 are counts on the first set of cores and mvc of cores 2 counts on the second set (Spearman rank correlation: *r* = 0.38, *P* = 0.48)

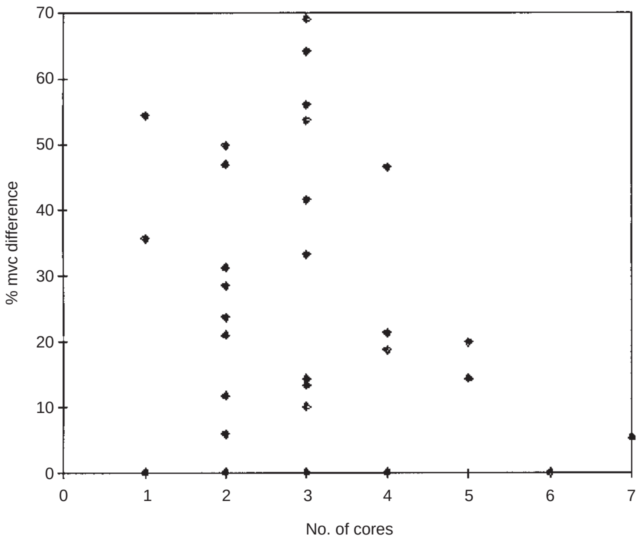

**Figure 7** Percentage difference in mvc between two tumour samples versus number of cores taken (Spearman rank correlation: *r* = 0.25, *P* > 0.05)

ranged from 1 to 6 (median: 3, mean: 2.95). Transverse sections of the same tumours were taken at surgery.

The relationship between counts of cores and subsequent tumour cross-sections is shown on Figure 5. Microvessel counts in core biopsies ranged from 12 to 25 (median: 16, mean: 17.1) and in later tumour cross-sections from 15 to 25 (median: 21, mean: 21.1). Although the range of mvc values in cores and subsequently excised material was similar, there was no significant correlation between them (*r* = –0.1). There was a significant difference in counts in the two sample types with values tending to be higher in tumour sections than core biopsies (*P* = 0.0065, paired Wilcoxon). This was reflected in 13 of 20 tumours (65%) in which counts were higher in tumour sections than cores, in four of 20 tumours (20%) counts were higher in core biopsies and identical values were obtained in three tumours (15%).

### Comparison between mvc of core biopsies at diagnosis and following surgical excision

Multiple core biopsies were taken of tumours in ten patients for diagnosis and were repeated immediately following surgical excision. The number of cores performed ranged from 1 to 7 (median: 3, mean: 3.05). Figure 6 illustrates the relationship between counts in cores at the two time points. Mvc in cores at diagnosis (mvc1) ranged from 10 to 22 (mean 16.4, median 16.5) and in cores at surgery (mvc2) ranged from 13 to 21 (mean: 17.4, median: 18). No significant association was found between counts in the pairs of cores (*r* = –0.38). Conversely, no significant difference in mvc was detected (*P* = 0.48), with counts being higher in later cores in five of 10 tumours (50%), higher in initial biopsies in four tumours (40%) and the same in one (10%).

### Correlation between number of cores performed and Codifference in mvc between cores and tumour section

Analysis was performed to determine whether there was a relationship between number of cores performed and the difference in mvc between cores and tumour sections. In 37 cases, cores and tumour section were available for comparison. Number of cores performed ranged from 1 to 7 (median: 3, mean: 3.13). Mvc difference was expressed as a percentage of total mvc count in the tumour section, and its relationship with number of cores is illustrated on Figure 7. No significant correlation was identified (*r* = –0.25, *P* > 0.05).

### DISCUSSION

The present study has attempted to assess the variation in assessment of angiogenesis which might be caused by (i) inherent variation in the method, (ii) tumour heterogeneity and (iii) previous surgical manipulation of tumour.

Variation in mvc between the same observers performed at different times was 11% in this study, with a highly significant correlation between the two sets of counts (*r* = 0.84, *P* < 0.001). Inter-observer variation was 15%, which is comparable to another study addressing the same issue (Martin et al, 1997*a*). The issues of observer variation have been raised in several studies as a possible reason for failure of angiogenesis assessment to provide useful prognostic information in some series of primary breast cancers (Van Hoef et al, 1993; Axelsson et al, 1995). In both studies, mvc was used as a categorized rather than a continuous variable (Van Hoef et al, 1993; Axelsson et al, 1995), which may result in loss of predictive power (Vermeulen et al, 1996). Another study addressing the issue of observer variation found that mvc provided independent prognostic information in a series of breast tumours when performed by experienced observers, but not by the least experienced observer (Vermeulen et al, 1997). The authors therefore recommended a period of training with an experienced observer (Vermeulen et al, 1997). In the present study, counts were performed by two observers, a pathologist and a surgeon, who had undertaken a period of training with an experienced observer before embarking on the study. Other methods have been suggested to improve objectivity of the counting method, such as use of a Chalkley eyepiece graticule, as described in the present

*© 1999 Cancer Research Campaign*

study, or computerized image analysis (Fox et al, 1995). In a recently published study comparing inter-observer variability using different counting methods, use of the Chalkley grid yielded low variability and was found to be more effective in reducing variation than selection of the same hot spot by different observers (Hansen et al, 1998). Reproducibility was further optimized by the use of a conference microscope, thereby eliminating subjectivity in hot spot selection and field sampling (Hansen et al, 1998). The low degree of inter-observer variability observed in the present study is likely to be related to the use of the Chalkley grid by two observers using a conference microscope.

Tumour heterogeneity in vasculature was investigated in the present study by comparing microvessel counts in core biopsies and tumour sections taken simultaneously. Whilst the range of counts was similar in the two groups, correlation between counts failed to reach statistical significant ($r = 0.44$, $P = 0.09$). Vessel counts in tumour sections tended to be higher than those in core biopsies. Heterogeneity of tumour vasculature between different areas of the same tumour is well-recognized (Weidner et al, 1991; Van Hoef et al, 1993) and is overcome to an extent by selection of three vascular ‘hot spots’. Alternatives to the original method proposed by Weidner have been suggested and may particularly benefit the less experienced observer. These are to perform counts in ten fields of high vascularity, taking the highest count as the mvc (Martin et al, 1997*a*), or to perform photomicrographs of the most intensely vascular areas and for individual observers to obtain counts from the photographs (Vermeulen et al, 1997). Core biopsies represent only a small cross-section of total tumour and are therefore less likely to include individual vascular ‘hot spots’ than a larger tumour section. Indeed, in some core biopsy specimens it was difficult to clearly identify three hot spots. The differences in vessel density in ‘hot spots’ found in core biopsies and tumour sections are illustrated on Figure 3, which clearly shows a lower density in the core biopsy compared with the tumour section. These observations were further borne out in the findings that counts in core biopsies were lower than those in tumour sections in 65% of tumours and higher in only 20%. Another study which addressed the role of core biopsies found a similar lack of correlation in mvc of cores and tumour sections, but found mvc to be higher in core biopsies than tumour sections in 61.2% of cases (Jacobs et al, 1998).

Problems with tumour heterogeneity are not unique to vascular assessments, but have been described for several other parameters in breast cancer. Significant variations in mitotic activity index assessed on haematoxylin and eosin sections (Jannink et al, 1996) and DNA cell cycle variables (Bergers et al, 1996) were found on assessment of multiple tumour sections. Conversely, levels of MIB-1 and oestrogen receptor expression were found to alter little in assessments of serial tumour sections (Jensen and Ladekarl, 1995).

The issue of the accuracy of core biopsies in providing prognostic information is important as such specimens are increasingly used in diagnosis and may provide the only pretreatment tissue available. Other studies have addressed similar issues for markers other than angiogenesis and reproducible results have been obtained for determinations of DNA ploidy, bcl-2, oestrogen receptor, c-erbB-2 and p53, which are dichotomously scored variables (Daidone et al, 1991; Jacobs et al, 1998). Jacobs et al failed to produce reproducible results for mvc and accuracy was not improved by increasing tumour area available for assessment by performing more core biopsies (Jacobs et al, 1998). This is in keeping with results from the present study, in which, whilst there is a suggestion from Figure 6 that difference in mvc may be reduced with an increasing number of core biopsies, this correlation failed to reach statistical significance.

Another possible reason for unreliability of sequential core biopsies in tumour vascular assessment is the likely induction of angiogenesis following initial biopsy as part of the normal process of wound healing (Folkman, 1971). Comparison of mvc in core biopsies and subsequent tumour sections and in sequential core biopsies with no intervening treatment failed to show any correlation (Figures 4 and 5), which may be due to inherent variability in vessels throughout the tumour and/or effects of previous surgical manipulation.

In summary, this study has addressed various practical issues regarding the methodology of tumour vascularity assessment by mvc. Intra- and inter-observer variation in counts were 11 and 15% respectively, which were similar to results of other studies. Tumour heterogeneity is a problem when adopting this method in small tumour biopsies, such as core biopsies, which may not contain vascular ‘hot spots’. In addition, angiogenesis takes place in conditions other than tumour growth, such as wound healing and is therefore likely to be stimulated by surgical manipulation of the tumour, affecting subsequent assessments. Results of the present study suggest that mvc on core biopsy specimens are unlikely precisely to represent overall tumour vascularity. Other markers of angiogenesis, such as angiogenic growth factor expression, which may be assessed in tumour or serum, may provide an alternative method of assessment. These may be less subject to such variations, as the methods involved are not dependent on selection of specific tumour areas for assessment.

### ACKNOWLEDGEMENTS

The authors would like to acknowledge the support of the Royal College of Surgeons of England and the Dunhill Medical Trust in funding work in angiogenesis at the Edinburgh Breast Unit, and of Dr R Elton, Statistical Consultant, for his statistical advice.

### REFERENCES

Axelsson K, Ljung EB-M, Moore DH II, Thor AD, Chew KL, Edgerton SM, Smith HS and Mayall BH (1995) Tumor angiogenesis as a prognostic assay for invasive ductal breast carcinoma. *J Natl Cancer Inst* **87**: 997–1008

Bergers E, van Diest PJ and Baak JP (1996) Tumour heterogeneity of cell cycle variables in breast cancer measured by flow cytometry. *J Clin Pathol* **49**: 931–937

Cordell JL, Falini B, Erber WN, Ghish AK, Abdulaziz Z, MacDonald S, Pulford KA, Stein H and Mason DY (1984) Immunoenzymatic labeling of monoclonal antibodies using immune complexes of alkaline phosphatase and monoclonal anti-alkaline phosphatase (APAAP complexes). *J Histochem Cytochem* **32**: 219–229

Costello P, McCann A, Carney DN and Dervan PA (1995) Prognostic significance of microvessel density in lymph node negative breast carcinoma. *Hum Pathol* **26**: 1181–1184

Daidone MG, Orefice S, Mastore M, Santoro G, Salvadori B and Silvestrini R (1991) Comparing core needle to surgical biopsies in breast cancer for cell kinetics and ploidy studies. *Breast Cancer Res Treat* **19**: 33–37

de Jong JS, van Diest DJ and Baak JP (1995) Heterogeneity and reproducibility of microvessel counts in breast cancer. *Lab Invest* **73**: 922–926

Folkman J (1971) Tumour angiogenesis: therapeutic implications. *N Engl J Med* **285**: 1182–1186

Folkman J (1990) What is the evidence that tumors are angiogenesis dependent? (editorial). *J Natl Cancer Inst* **82**: 4–6

Folkman J (1994) Angiogenesis and Breast Cancer (editorial). *J Clin Oncol* **12**: 441–443

*© 1999 Cancer Research Campaign*

Fox SB, Leek RD, Weekes MP, Whitehouse RM, Gatter KC and Harris AL (1995) Quantitation and prognostic value of breast cancer angiogenesis: comparison of microvessel density, Chalkley count, and computer image analysis. *J Pathol* **177**: 275–283

Gasparini G, Weidner N, Bevilacqua P, Maluta S, Dalla Palma P, Caffo O, Barbareschi M, Boracchi P, Marubini E and Pozza F (1994) Tumor microvessel density, p53 expression, tumor size, and peritumoral lymphatic vessel invasion are relevant prognostic markers in node-negative breast carcinoma. *J Clin Oncol* **12**: 454–466

Hansen S, Grabau DA, Rose C, Bak M and Sorensen FB (1998) Angiogenesis in breast cancer: a comparative study of the observer variability of methods for determining microvessel density. *Lab Invest* **78**: 1563–1573

Horak ER, Leek R, Klenk N, LeJeune S, Smith K, Stuart N, Greenall M, Stepniewska K and Harris AL (1992) Angiogenesis, assessed by platelet/endothelial cell adhesion molecule antibodies, as indicator of node metastases and survival in breast cancer. *Lancet* **340**: 1120–1124

Jacobs TW, Siziopikou KP, Prioleau JE, Raza S, Baum JK, Hayes DF and Schnitt SJ (1998) Do prognostic marker studies on core needle biopsy specimens of breast carcinoma accurately reflect the marker status of the tumour? *Modern Pathol* **11**: 259–264

Jannink I, Risberg B, Van Diest PJ and Baak JP (1996) Heterogeneity of mitotic activity in breast cancer. *Histopathology* **29**: 421–428

Jensen V and Ladekarl M (1995) Immunohistochemical quantitation of oestrogen receptors and proliferative activity in oestrogen receptor positive breast cancer. *J Clin Pathol* **48**: 429–432

Martin L, Green B, Renshaw C, Lowe D, Rudland P, Leinster SJ and Winstanley J (1997*a*) Examining the technique of angiogenesis assessment in invasive breast cancer. *Br J Cancer* **76**: 1046–1054

Martin L, Holcombe C, Green B, Leinster SJ and Winstanley J (1997*b*) Is a histological section representative of whole tumour vascularity in breast cancer? *Br J Cancer* **76**: 40–43

Minkowitz S, Moskowitz R, Khafif RA and Alderete MN (1986) TRU-CUT needle biopsy of the breast. An analysis of its specificity and sensitivity. *Cancer* **57**: 320–323

Parums DV, Cordell JL, Micklem K, Heryet AR, Gatter KC and Mason DY (1990) JC70: a new monoclonal antibody that detects vascular endothelium associated antigen on routinely processed tissue sections. *J Clin Pathol* **43**: 752–757

Van Hoef MEHM, Knox WF, Dhesi SS, Howell A and Schor AM (1993) Assessment of tumour vascularity as a prognostic factor in lymph node negative breast cancer. *Eur J Cancer* **29A**: 1141–1145

Vermeulen PB, Gasparini G, Fox SB, Toi M, Martin L, McCulloch P, Pezzella F, Viale G, Weidner N, Harris AL and Dirix LY (1996) Quantification of angiogenesis in solid human tumours: an international consensus on the methodology and criteria for evaluation. *Eur J Cancer* **32A**: 2474–2484

Vermeulen PB, Libura M, Libura J, O'Neill PJ, van Dam P, Van Marck E, Van Oosterom AT and Dirix LY (1997) Influence of investigator experience and microscopic field size on microvessel density in node-negative breast carcinoma. *Br Cancer Res Treat* **42**: 165–172

Weidner N, Semple JP, Welch WR and Folkman J (1991) Tumor angiogenesis and metastasis – correlation in invasive breast carcinoma. *N Engl J Med* **324**: 1–8

Weidner N, Folkman J, Pozza F, Bevilacqua P, Allred EN, Moore DH, Meli S and Gasparini G (1992) Tumor angiogenesis: a new significant and independent prognostic indicator in early-stage breast carcinoma. *J Natl Cancer Inst* **84**: 1875–1887

*© 1999 Cancer Research Campaign*

## References

[PDF_00439] Axelsson K., Ljung B. M., Moore D. H., Thor A. D., Chew K. L., Edgerton S. M., Smith H. S., Mayall B. H. (1995). Tumor angiogenesis as a prognostic assay for invasive ductal breast carcinoma.. J Natl Cancer Inst.

[PDF_00442] Bergers E., van Diest P. J., Baak J. P. (1996). Tumour heterogeneity of DNA cell cycle variables in breast cancer measured by flow cytometry.. J Clin Pathol.

[PDF_00445] Cordell J. L., Falini B., Erber W. N., Ghosh A. K., Abdulaziz Z., MacDonald S., Pulford K. A., Stein H., Mason D. Y. (1984). Immunoenzymatic labeling of monoclonal antibodies using immune complexes of alkaline phosphatase and monoclonal anti-alkaline phosphatase (APAAP complexes).. J Histochem Cytochem.

[PDF_00450] Costello P., McCann A., Carney D. N., Dervan P. A. (1995). Prognostic significance of microvessel density in lymph node negative breast carcinoma.. Hum Pathol.

[PDF_00453] Daidone M. G., Orefice S., Mastore M., Santoro G., Salvadori B., Silvestrini R. (1991). Comparing core needle to surgical biopsies in breast cancer for cell kinetic and ploidy studies.. Breast Cancer Res Treat.

[PDF_00462] Folkman J. (1994). Angiogenesis and breast cancer.. J Clin Oncol.

[PDF_00458] Folkman J. (1971). Tumor angiogenesis: therapeutic implications.. N Engl J Med.

[PDF_00460] Folkman J. (1990). What is the evidence that tumors are angiogenesis dependent?. J Natl Cancer Inst.

[PDF_00466] Fox S. B., Leek R. D., Weekes M. P., Whitehouse R. M., Gatter K. C., Harris A. L. (1995). Quantitation and prognostic value of breast cancer angiogenesis: comparison of microvessel density, Chalkley count, and computer image analysis.. J Pathol.

[PDF_00470] Gasparini G., Weidner N., Bevilacqua P., Maluta S., Dalla Palma P., Caffo O., Barbareschi M., Boracchi P., Marubini E., Pozza F. (1994). Tumor microvessel density, p53 expression, tumor size, and peritumoral lymphatic vessel invasion are relevant prognostic markers in node-negative breast carcinoma.. J Clin Oncol.

[PDF_00475] Hansen S., Grabau D. A., Rose C., Bak M., Sørensen F. B. (1998). Angiogenesis in breast cancer: a comparative study of the observer variability of methods for determining microvessel density.. Lab Invest.

[PDF_00478] Horak E. R., Leek R., Klenk N., LeJeune S., Smith K., Stuart N., Greenall M., Stepniewska K., Harris A. L. (1992). Angiogenesis, assessed by platelet/endothelial cell adhesion molecule antibodies, as indicator of node metastases and survival in breast cancer.. Lancet.

[PDF_00482] Jacobs T. W., Siziopikou K. P., Prioleau J. E., Raza S., Baum J. K., Hayes D. F., Schnitt S. J. (1998). Do prognostic marker studies on core needle biopsy specimens of breast carcinoma accurately reflect the marker status of the tumor?. Mod Pathol.

[PDF_00486] Jannink I., Risberg B., Van Diest P. J., Baak J. P. (1996). Heterogeneity of mitotic activity in breast cancer.. Histopathology.

[PDF_00488] Jensen V., Ladekarl M. (1995). Immunohistochemical quantitation of oestrogen receptors and proliferative activity in oestrogen receptor positive breast cancer.. J Clin Pathol.

[PDF_00491] Martin L., Green B., Renshaw C., Lowe D., Rudland P., Leinster S. J., Winstanley J. (1997). Examining the technique of angiogenesis assessment in invasive breast cancer.. Br J Cancer.

[PDF_00494] Martin L., Holcombe C., Green B., Leinster S. J., Winstanley J. (1997). Is a histological section representative of whole tumour vascularity in breast cancer?. Br J Cancer.

[PDF_00497] Minkowitz S., Moskowitz R., Khafif R. A., Alderete M. N. (1986). TRU-CUT needle biopsy of the breast. An analysis of its specificity and sensitivity.. Cancer.

[PDF_00500] Parums D. V., Cordell J. L., Micklem K., Heryet A. R., Gatter K. C., Mason D. Y. (1990). JC70: a new monoclonal antibody that detects vascular endothelium associated antigen on routinely processed tissue sections.. J Clin Pathol.

[PDF_00504] Van Hoef M. E., Knox W. F., Dhesi S. S., Howell A., Schor A. M. (1993). Assessment of tumour vascularity as a prognostic factor in lymph node negative invasive breast cancer.. Eur J Cancer.

[PDF_00507] Vermeulen P. B., Gasparini G., Fox S. B., Toi M., Martin L., McCulloch P., Pezzella F., Viale G., Weidner N., Harris A. L. (1996). Quantification of angiogenesis in solid human tumours: an international consensus on the methodology and criteria of evaluation.. Eur J Cancer.

[PDF_00512] Vermeulen P. B., Libura M., Libura J., O'Neill P. J., van Dam P., Van Marck E., Van Oosterom A. T., Dirix L. Y. (1997). Influence of investigator experience and microscopic field size on microvessel density in node-negative breast carcinoma.. Breast Cancer Res Treat.

[PDF_00518] Weidner N., Folkman J., Pozza F., Bevilacqua P., Allred E. N., Moore D. H., Meli S., Gasparini G. (1992). Tumor angiogenesis: a new significant and independent prognostic indicator in early-stage breast carcinoma.. J Natl Cancer Inst.

[PDF_00516] Weidner N., Semple J. P., Welch W. R., Folkman J. (1991). Tumor angiogenesis and metastasis--correlation in invasive breast carcinoma.. N Engl J Med.

[PDF_00456] de Jong J. S., van Diest P. J., Baak J. P. (1995). Heterogeneity and reproducibility of microvessel counts in breast cancer.. Lab Invest.

